# Effect of Nanostructures on the Properties of Glass Ionomer Dental Restoratives/Cements: A Comprehensive Narrative Review

**DOI:** 10.3390/ma14216260

**Published:** 2021-10-21

**Authors:** Faiza Amin, Sehrish Rahman, Zohaib Khurshid, Muhammad Sohail Zafar, Farshid Sefat, Naresh Kumar

**Affiliations:** 1 Science of Dental Materials Department, Dow Dental College, Dow University of Health Sciences, Karachi 74200, Pakistan; faiza.ameen@duhs.edu.pk; 2 Science of Dental Materials Department, Dr. Ishrat Ul Ebad Khan Institute of Oral Health Sciences, Dow University of Health Sciences, Karachi 74200, Pakistan; dr.sehrish.rahman@gmail.com (S.R.); kumar.naresh@duhs.edu.pk (N.K.); 3 Department of Prosthodontics and Dental Implantology, College of Dentistry, King Faisal University, Al-Ahsa 31982, Saudi Arabia; 4 Department of Restorative Dentistry, College of Dentistry, Taibah University, Al Madinah, Al Munawwarah 41311, Saudi Arabia; MZAFAR@taibahu.edu.sa; 5 Department of Dental Materials, Islamic International Dental College, Riphah International University, Islamabad 44000, Pakistan; 6 Department of Biomedical and Electronics Engineering, School of Engineering, University of Bradford, Bradford BD7 1DP, UK; f.sefat1@bradford.ac.uk

**Keywords:** nanostructures, nanotechnology, glass ionomer cement, mechanical properties, antibacterial activity, fluoride release

## Abstract

Overall perspective of nanotechnology and reinforcement of dental biomaterials by nanoparticles has been reported in the literature. However, the literature regarding the reinforcement of dental biomaterials after incorporating various nanostructures is sparse. The present review addresses current developments of glass ionomer cements (GICs) after incorporating various metallic, polymeric, inorganic and carbon-based nanostructures. In addition, types, applications, and implications of various nanostructures incorporated in GICs are discussed. Most of the attempts by researchers are based on the laboratory-based studies; hence, it warrants long-term clinical trials to aid the development of suitable materials for the load bearing posterior dentition. Nevertheless, a few meaningful conclusions are drawn from this substantial piece of work; they are as follows: (1) most of the nanostructures are likely to enhance the mechanical strength of GICs; (2) certain nanostructures improve the antibacterial activity of GICs against the cariogenic bacteria; (3) clinical translation of these promising outcomes are completely missing, and (4) the nanostructured modified GICs could perform better than their conventional counterparts in the load bearing posterior dentition.

## 1. Introduction

The glass ionomer cement (GIC) is a translucent, water-based cement invented in 1972 by Wilson and Kent. The terminology is based on fluorosilicate glass and polyacrylic acid (PAA) as its original components [1]. The reaction occurs by acid-base interaction between ion leachable fluorosilicate glass powder and aqueous solution of PAA [2]. The bioactivity of GIC is possible due to their capability to supply the therapeutic ions when PAA reacts with ion leachable glasses [3,4,5]. Hydrophilic characteristics of PAA in these cements increase the bioactivity compared to resin-based materials [6]. After mixing the base (powder) and acid (liquid), the cement hardens in 2–10 min [7]. GlCs bond chemically via a carboxyl group from cement itself to the dentin or enamel of the tooth structure through calcium. In addition, these cements release fluoride ions that induce anticariogenic property for a fairly long period [8,9]. Many mechanisms are involved in the anticariogenic effects of fluoride, including the inhibition of bacterial growth and metabolism in the oral cavity, decreased demineralization, increased remineralization of the dental hard tissues and inhibition of pellicle and plaque formation on the tooth surface. It is assumed that caries formation is restricted through all these mechanisms after the release of fluoride from GICs [10]. Moreover, their stability in an aqueous environment, similar coefficient of thermal expansion to the tooth structure, biocompatibility, good marginal seal properties and high retention have led to their extensive use as cavity bases and liners, direct filling materials and luting materials [11,12].

Despite many advantages, the GICs are not widely used as permanent restorative materials for stress bearing areas because of their poor mechanical properties. During high masticatory stresses, the material is likely to fail due to its poor fracture toughness, tensile strength, wear resistance and hardness [9]. To enhance the physical and mechanical properties of GICs without compromising the biological or handling properties, many modifications have endeavored to the inorganic component of GICs. In this regard, fibers, metals, and other nonreactive fillers have been assessed. The most challenging task in this context is to achieve adhesion between the cement matrix and reinforcing agents. Moreover, to strengthen the GICs, various chemical modifications have been explored [13]. One method is that high powder to liquid ratio was achieved by incorporating glass particles with controlled particle sizes. This approach resulted in high-viscosity GICs. The properties of these high viscosity materials were superior compared to the conventional GICs [14]. Other approaches have involved the addition of aluminosilicate fibres, amalgam alloys, carbon, hydroxyapatite (HA) and stainless-steel particles [15,16,17]. GICs modified by incorporating nanostructures exhibited fewer air voids and internal microcracks. In addition, apparently modified materials are easier to handle than unmodified cements, which resulted in greater strengths in compression [18]. To modify the chemical, biological, and physical properties of dental restorative materials, manufacturers incorporated a myriad of nanoparticles (NPs), making them innovative [19]. Over the past decade, the inclination of researchers towards the field of nanotechnology has been reflected by increased number of patent applications. Nanostructures incorporated in dental biomaterials enhanced their properties [20]. A brief review of nanostructures added in various dental materials and their characteristics and applications is presented in Table 1.

Due to the distinctive properties, GICs have been widely used in dentistry for more than four decades. Common dental applications of GICs include permanent restorative materials in pediatric dentistry, liners, bases and fissure sealants. One of the most exclusive applications of GICs in orthodontics includes bonding of orthodontic brackets and bands with the tooth structure [1]. In addition, GICs exhibited good clinical outcomes and high durability when used for Atraumatic Restorative Treatment (ART) [49,50]. However, their mechanical characteristics are not adequate to sustain the masticatory forces as described above; hence, to improve their strength, several modifications have been investigated. For example, amalgam alloys, aluminosilicate fibres, carbon, fillers, HA powder and stainless steel have been added to strengthen the GICs [51]. Moreover, in dentistry, the addition of nanostructures has become an important field of research. Various types of nanomaterials including ceramic, metal or polymers, are added in GICs to improve the mechanical properties [17,52,53,54,55,56,57,58,59,60,61,62,63,64,65,66,67,68,69,70,71,72,73,74]. The properties of GICs have been successfully enhanced by incorporating metal alloys at the nanoscale level (i.e., silver–tin or silver–palladium/titanium) into glass powder [75,76]. Similarly, nanoionomers have been amalgamated with GICs to improve the surface properties. Oxman and colleagues [77] compared two fluoroalumiosilicate resin modified glass ionomer cements (RMGIs) and a nanohybrid composite with nanoionomeric hybrid resin-modified glass ionomer (NHRMGI) and reported a significantly higher gloss with the material reinforced with the nanostructures. Wear rates for nanohybrid were significantly higher than other RMGIs [77]. Inorganic silica nanofillers (~40 nm size) into the liquid of GIC is another recent development that increases the strength of the polymer matrix. This reinforcement decreased the initial setting time, improved the wear resistance, better resistance to dissolution and disintegration. The reinforced ionomers retained a polished surface for a longer period of time compared to the conventional GICs. In addition, these newer cements were also far better in terms of optical properties and translucency [78]. Friedl et al. [79] incorporated nanofillers into the GICs and concluded that GICs with nanofillers can be used for posterior fillings due to improved mechanical properties [79]. Nano-hydroxyapatite (Nano-HA) and nanoflouroapatite (Nano-FA) were added to conventional GICs [80] that improved mechanical properties such as biaxial flexural strength, compressive strength, and diametral tensile strength compared to the conventional GICs [81].

Plenty of research has been conducted where researchers investigated an overall perspective of nanotechnology and reinforcement of dental biomaterials by NPs. However, the information regarding the reinforcement of GICs after incorporating various nanostructures is still lacking. There is a dearth of literature as none of the review discuss all of the nanostructures, along with their properties, in a single paper. So, to bridge the gap of knowledge about the incorporation of NPs and their impact on the GICs, different approaches aiming to contribute to this paper felt indispensable. Therefore, the aim of this review is to highlight the impact of different nanostructures on the various properties of GICs, which may help researchers and industry while designing the clinical trials and translation of these materials for restorative dentistry applications. This is of the kind of review which highlights incorporation of almost all the nanostructures structures and studies its mechanical, physical, biological and toxicological properties and their impact on the GICs. 

## 2. Structure and Composition of Conventional GICs

**Figure 1:**
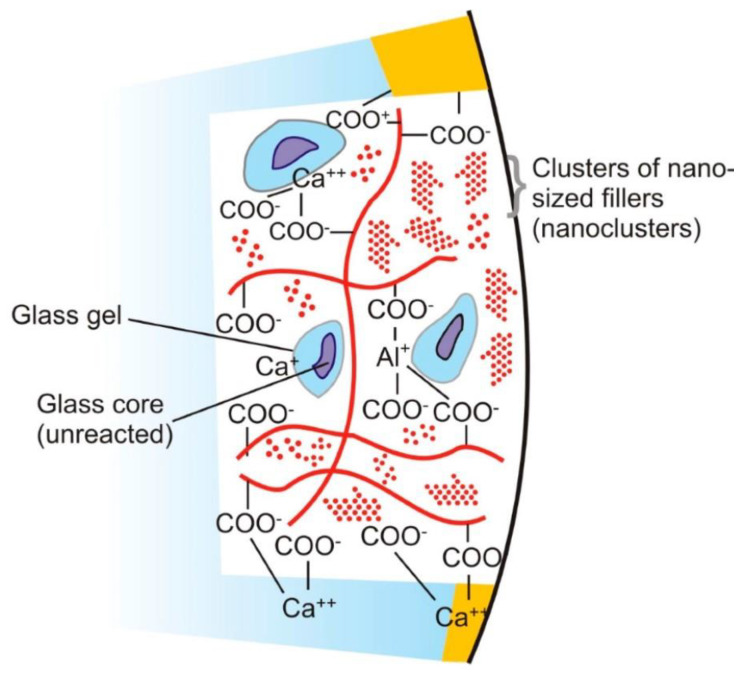
Diagram represents the structure of glass ionomer cement and its chemical bonding with tooth structure. Adopted from reference [24].

**Figure 2:**
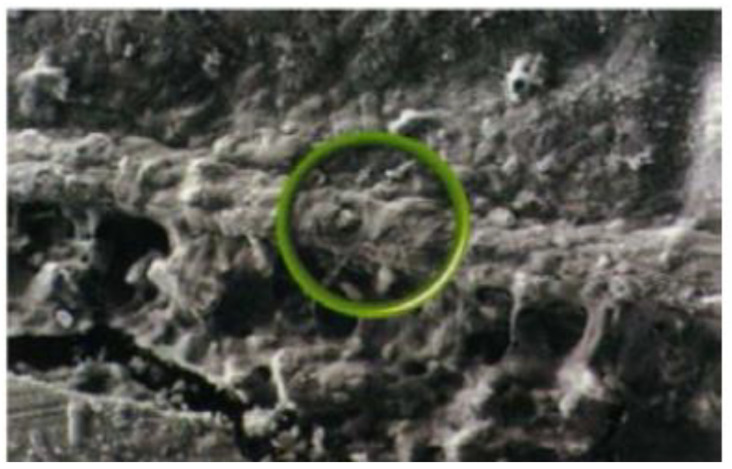
SEM Analysis representing Interfacial ion-exchange layer formed between tooth surface (above) and glass-ionomer cement (below). The circle indicates part of the ion-exchange layer. Reprinted from ref. [1].

The powder component of GICs (fluoroaluminosilicate) has the capability to leach ions upon reaction with PAA. Alumina (Al_2_O_3_), aluminium phosphate (AlPO_4_), calcium fluoride (CaF_2_), cryolite (Na_3_AlF_6_), sodium fluoride (NaF) and silica (SiO_2_) are the basic constituents of the powder [82]. Currently, the liquids are homo and copolymers of itaconic, maleic, or tricarboxylic acids. Formerly, PAA (~40% to 50%) were used but had a short shelf life because of high viscosity and gelation tendency. The gelation occurs due to excessive intermolecular hydrogen bonding. The liquid also contains tartaric acid that controls the setting characteristics [83]. GICs comprise an aluminosilicate network which is the three-dimensional structure. In the glass matrix, aluminum ions (Al) play a dual role of network forming and network dwelling ions, whereas silicone (Si) ions exist in interstices formed by four oxygen anions. Reaction initiates when loosely bound negative charges from the glass are attacked by carboxylic acid. Carboxylic acid attack at the Al ions network sites results in the disturbance in the three-dimensional matrix. After the attack, ionic bonds with polymers are formed when Al ions and other ions are released. The rate of setting reaction in GICs is controlled by Al/Si ratio [84,85] (Figure 1). This process involves formation of ionic bonds between calcium ions on the tooth surface and carboxylate groups on the polyacid molecules [1]. The ratio of counter ions should be close to the Al/Si ratio so that proper glass network is formed prior to mixing of acidic polymer into the glass. Reaction is initiated when loosely bound negative charges from the glass attack by carboxylic acid. Carboxylic acid attack at the Al ions network sites resulting in the disturbance in the three-dimensional matrix. After the attack, ionic bonds with polymers are formed when Al ions and other ions are released. Al/Si ratio controlled the rate of setting reaction in GICs. The ratio of Al_2_O_3_ to SiO_2_ (1:2 or more by mass) is critical for the cement’s accurate reactivity and hydrolytic stability. Over time, an ion-exchange layer is created through a diffusion process in which ions from the tooth and GIC move into the interfacial zone, establishing a strong chemical bond between tooth and the cement. In Figure 2, scanning electron microscopy exhibited the interfacial layer formed between glass-ionomer cement, Fuji IX (GC, Tokyo, Japan), and the tooth [1]. Regarding the setting characteristics and properties of GICs, the cements set within 2–3 min by an acid base reaction. Ion concentrations other than Al and Si also play a major role in the setting of GICs. Numerous spectroscopic techniques, such as 13C NMR, infrared and Fourier transform infrared (FTIR), are used to investigate the setting characteristics. These techniques concluded that the setting of GICs take place through the diffusion-controlled process in two steps. The first step is the formation of ionic cross links, which is responsible for the initial hardening of the cement. The second step is the maturation step, which is a continuous and ongoing process for a day [1]. To enhance the aesthetics property and the transparency, the Al_2_O_3_/SiO_2_ ratio has been changed along with reducing the amount of CaF_2_. To decrease the melting point, CaF_2_ was added as a flux. The radiopacity is achieved by the incorporation of barium (Ba), strontium (Sr), lanthanum (La), or zinc (Zn) [1,24].

## 3. Rationale and Significance of the Review

Richard Feynman, a physicist, firstly introduced the concept of nanotechnology in 1959 [86] and it is considered as the unique route by which researchers can operate materials at molecular or atomic levels below 100 nm scale. Nanoparticles exhibit exceptional biological, physical and chemical properties in terms of biocompatibility, biodegradability, crystallinity, particle size distribution and greater surface areas to volume ratio as compared to the normal/traditional particles. As a result, their applications are well evident in the fields of medicine and dentistry in order to develop new devices and materials with superior/novel properties [87,88]. Hence, a positive effect of NPs on the clinical performance of GICs may be expected.

In recent reviews, an overall perspective of nanotechnology and reinforcement of dental biomaterials by NPs has been presented [18,89,90,91,92,93,94,95,96]. However, there is a dearth of literature observed regarding the reinforcement of dental biomaterials after incorporating various nanostructures. In the present review, we have addressed nano dentistry related to most of the current developments of GICs after incorporation with (i) metallic nanostructures (e. g., copper and silver); (ii) polymeric nanostructures (e.g., chitosan and cellulose,); (iii) nano-based structures having inorganic component (e.g., hydroxyapatite and zirconia); (iv) carbon-based nanostructures (e.g., carbon nanotubes and graphene) [18,89,90,91,92,93,94,95,96]. This review narrates the types, application, and impact of various nanostructures incorporated in GICs. Previously, most of the work was focused mainly on NPs in different dental biomaterials, but this is one of the kinds of review which highlights incorporation of almost all the structures at the nanoscale level and studies the impact of these nanostructures, including NPs on the GICs.

## 4. Specific Nanostructures Incorporated in GICs

Various types of nanostructures added to GICs can be classified under three main categories based on either their origin, (a. natural b. artificial), dimensions (zero-dimensional nanostructures, one-dimensional or nanorods, and two-dimensional or thin films) or structural configuration (carbon-based, metal-based, polymeric-based and inorganic-based nanostructures [97,98]. The advantages, limitations and clinical significance of each nanostructure are presented in Table 2.

### 4.1. Metal-Based Nanostructures

Metals and metal oxides are used to synthesize NPs of sizes as low as 10 to 100 nm [99]. These metallic NPs are of different shapes like spherical and cylindrical, having amorphous and crystalline structures. These metallic NPs have unique characteristics; for instance, surface charge density, variable pore size, high surface area to volume ratio and color reactivity [100] and exhibit different physical, chemical, and mechanical properties as compared to their bulk counterparts. Metal and metal oxide NPs are mostly prepared using addition of either reducing or oxidizing agents. In GICs, the effect of nanostructures based on pure metals, as well as metal oxide, has been investigated and is described separately as follows:

#### 4.1.1. Pure Metal-Based Nanostructures

##### Silver

Silver has been investigated in numerous areas of medicine and dentistry because of its broad spectrum of antimicrobial activity against fungi, protozoa, some viruses, and Gram negative and Gram-positive bacteria [101,102,103]. Silver has been used for many biomedical applications such as bone prostheses, cardiac devices, catheters, reconstructive orthopaedic surgery, surgical appliances, water purification and wound care [104,105,106]. The degree of antibacterial, antifungal or antiviral response of these silver ions is dependent upon the number of ions being released and the extent of their interaction with the respective cell membranes. Recently, silver has been used as an elementary and an ionized material in the form of silver zeolites or NPs through nanotechnology, which made it possible better to explore the antimicrobial properties of silver [107]. NPs are smaller than 100 nm in size, which are insoluble clusters [108]. These particles have a higher surface area to volume ratio and therefore display more potent antibacterial activity [109].

Its mechanism of antibacterial action is that it increases the permeability of the bacterial cell wall, which in turn causes its loss of integrity. In addition, silver ions penetrate and adhere to the bacterial cell wall resulting in the loss of DNA replication ability and inactivation of essential enzymes of bacteria [110]. Moreover, in a low concentration, silver NPs are biocompatible [111]. The antibacterial efficacy for silver NPs is twenty-five times higher than chlorhexidine [112]. For the treatment of carious lesions, it is recommended to use GICs containing antibiotics so that the total number of viable cariogenic bacteria can be reduced. However, the addition of these antibiotics should not compromise the mechanical properties and biocompatibility [113,114]. In a study, cytotoxicity of the cements has been evaluated and found that cytotoxicity of GICs was not affected when these cements were incorporated with variable concentrations of sliver NPs [115].

Effects of the incorporation of various nanostructures on the mechanical strength of GICs are presented in Table 3. Jowkar et al. [116] investigated mechanical properties and dentin micro-shear bond strength of neat GIC in comparison to the silver NPs modified GICs. The GICs that contain silver NPs showed significantly higher compressive strength, surface microhardness, and dentin micro-shear bond strength compared to the conventional GICs [116]. To formulate an experimental GIC with antibacterial effects, researchers employed a single step photoreduction of silver NPs in a polyacrylate solution. In was concluded that silver NPs formed by one-step preparation in polyacrylate solution permitted the fabrication of highly bioactive cements that can be used for posterior and permanent dental restorations [117]. The potential toxic effect of silver NPs on pulp cells— namely, odontoblasts (MDPC-23 cells)—was investigated in a study in which authors evaluated the cytotoxicity of conventional and resin-modified GICs with and without addition of silver NPs. The silver NPs were added at two different concentrations (0.1 wt% and 0.2 wt%). Cells viability was evaluated by MTT and Trypan Blue assays. The silver NPs did not affect the cytotoxicity of the GICs, and resin modified GICs [118]. Porter et al. [119] investigated the antibacterial and mechanical properties of reinforced GICs after the incorporation of silver NPs. To evaluate biofilm accumulation of *Streptococcus mutans* at (72 h) viability stain with confocal laser scanning microscopy was used. In addition, the compressive, flexural strength and color stability of the specimens was evaluated in comparison to the unmodified samples. They also investigated leaching out of silver ions from silver NPs modified GIC specimens using inductively coupled plasma-mass spectrometry. Not only did the silver NPs modified GICs hold on to mechanical properties equal to or even higher to that of unmodified GICs, but they also exhibited significant antibiofilm activity (Table 4). Thus, it can be said that this formulation of modified GICs can lead to reduction of secondary caries by reducing the bacterial colonization around the restorations [119]. In addition, their positive influence on the strength and surface hardness of GICs suggest an enhanced survival rate during the clinical life.

##### Copper

Currently, GICs with high viscosity, long-term fluoride release potential, acceptable biocompatibility and tooth-matching thermal expansion are advocated according to the clinical guidelines [120]. Despite the presence of fluoride in GICs, their total released amount is not enough to achieve anticariogenic properties [121]. As a result, secondary caries remains an unsolved dilemma. To deal with this issue, various antibacterial additives, namely natural products, for example, metals, quaternary ammonium salts [122], antiseptics [123] and propolis [124,125] have been proposed to alter the GIC preparations.

In recent years, the antibacterial characteristics of the copper nanometric particles have also been explored [126]. A decrease in particle size provides the greater surface area for the interaction of bacterial area. The antibacterial efficacy of the copper oxide (Cu_2_O) against Gram-negative and positive bacteria is also well evident in the literature. Aguilar-Perez et al. [127] produced copper nanoparticles using a green method and blended with GIC. The addition of copper NPs (2–4 wt%) highlighted the antibacterial activity of GIC against the strains of anaerobic bacteria including *Streptococcus mutans* and *Streptococcus sanguinis* (Table 4). A small amount of copper NPs in GICs may provide a suitable antibacterial activity to prevent the secondary caries. However, further studies are warranted to assess the effects of such an addition on the mechanical profile of GICs.

#### 4.1.2. Metal Oxide—Based Nanostructures

##### Magnesium Oxide

Magnesium oxide (MgO) NPs possess antibacterial properties and are considered suitable candidates for biomedical science [128]. These particles can be easily synthesized from the economic precursors [129]. The MgO NPs manufactured by chemical processing has shown antibacterial activity against *Streptococcus mutans* in recent studies [130,131]. MgO NPs and their biodegradable products are highly biocompatible, and the US Food and Drug Administration has categorized them under harmless materials [132].

Noori and Kareem [133] incorporated the MgO NPs in GIC powder (Ketac Molar Easymix) at various concentrations and evaluated their antibacterial activity against *Streptococcus mutans* and *Streptococcus sobrinus* using biofilm-CFU counting assays and agar disk diffusion method. The addition of 1% MgO NPs in GIC was found effective against both microorganisms in terms of the antibacterial activity **(Table 4**). The authors proposed that the addition of MgO NPs in GIC could be considered whilst the development of dental restorative cements.

After gaining positive antibacterial effect of MgO NPs in GIC, the same researchers [134] further analyzed the setting characteristics, mechanical properties and adhesive properties of the modified GICs. The incorporation of MgO NPs in GIC led to an increase in the setting time of the cement. Moreover, GICs with 1% MgO NPs exhibited greater compressive, diametral tensile and shear bond strength values (Table 3). The aforementioned findings indicate the potential of MgO NPs in terms of antibacterial, as well as mechanical performance; hence, likelihood of the development of improved GIC restorative materials is high. Moreover, the synthesis of MgO NPs from the economic precursors may aid in the production of cost-effective materials.

##### Titanium Dioxide

In the field of medicine and dentistry, titanium dioxide NPs (TiO_2_ NPs) are frequently used [135] due to their significant antibacterial activity and high surface energy. These NPs have good anti-adhesive properties against *Streptococcus mutans* and produce free radicals that damage the oxidative process of the cell wall of a microorganism [136]. Additionally, their antibacterial properties are better than chlorhexidine and used for preventing white spot formation during orthodontic treatment [137].

E. Elsaka et al. [138] performed tests to study the antibacterial and mechanical properties of conventional GICs after adding TiO_2_ NPs to 3%, 5% and 7wt% concentration. The control group for this study was unmodified GIC. Using a universal testing machine, compressive strength, fracture toughness, flexural strength and microtensile bond strength were assessed. Vickers microhardness tester was used to determine surface microhardness. Setting time was investigated according to ISO standard. A direct contact test was used to evaluate the antibacterial activity against *Streptococcus mutans*. The amount of fluoride release was evaluated both for modified and control GICs. The fracture toughness, flexural strength, and compressive strengths of GICs were improved with the addition of 3 wt% and 5 wt% TiO_2_ NPs (Table 3), whereas a marked reduction in the mechanical properties was noted for GICs having 7 wt% TiO_2_ NPs (Table 3). Surface microhardness was decreased with 5% and 7 wt% TiO_2_ NPs. GICs containing TiO_2_ NPs decreased the setting time, but the values were still within the confines of ISO specified limits. The bond strength with dentine after the incorporation of TiO_2_ NPs to the conventional GIC did not get compromised as well as a modification with TiO_2_ NPs did not reduce the fluoride release. It was found that TiO_2_ NPs possessed a solid antibacterial effect (Table 4). The authors concluded their finding as enhanced mechanical and antibacterial properties were obtained when GIC was modified with 3 wt% TiO_2_ NPs, and therefore it can serve as an effective direct restorative material. This formulation was recommended for potential restoration applications in higher masticatory load-bearing sites [138]. Very recently, to overcome the weakness of the GICs, researchers incorporated Al2O_3,_ ZrO_2_ and TiO_2_ NPs into two different commercially available GICs. The group without NPs was the control group of the study. Three percentages of NPs were added (2, 5 or 10 wt%) to conventional GIC. The compressive strengths were measured using a universal testing machine. Type of the fracture was evaluated through scanning electron microscopy (SEM) and the morphology of fractured surfaces was also analyzed. Inductively coupled plasma-optical emission spectrometry (ICP-OES) was used to identify the components leached into the storage solutions. The authors identified an increased compressive strength with the addition of ZrO_2_ and especially TiO_2_ NPs, whereas the cement structure was weakened after the incorporation of Al_2_O_3_ NPs. However, these modified cements were considered suitable for clinical use as they did not leach noticeable levels of ions (Al, Zr or Ti). [139]. To assess anti-adherent and antibacterial properties, TiO_2_ NPs were used as surface treatment of orthodontic wires. Researchers found that uncoated wires exhibited slight increase in bacterial adhesion compared with the coated wires against *S. mutans*. Antibacterial effects against *P. Gingivalis* showed initial antibacterial activity in the first 30 minutes, and then it was stable. It is normal to develop dental plaque during orthodontic treatment that can be prevented by the surface treatment of orthodontic wires with TiO_2_ NPs [140]. In a study conducted by Özyıldız F et al. [141] researchers evaluated the antibacterial effect of ceramic brackets coated with TiO_2_ against *S. mutans and C. albicans* illuminated with UV. Sol gel dip-coating method was used in the study. It was found that the number of attached cells reduced after TiO_2_ film coating [141]. These findings about the influence of TiO_2_ clearly indicate their great potential in the fields of restorative dentistry and orthodontics.

##### Zinc Oxide

Zinc oxide (ZnO) is multifunctional and semiconductor filler that provides many beneficial properties including biocompatibility, non-toxicity to cells, chemical stability, cost-effectiveness, and bactericidal activity [142,143]. Additionally, due to its antibacterial and antimicrobial characteristics against *Streptococcus mutans* and periodontal pathogens, ZnO is widely used in the preparations of toothpaste and mouth rinses [144]. When using nanosized ZnO, its advantages become more noticeable because more significant interaction of particles and organic molecules can be observed due to higher surface-to-volume ratio [143,144]. When GICs are incorporated with ZnO particles, researchers demonstrate their physico-mechanical and antibacterial properties [145]. Their antibacterial activity is attributed to the adverse changes resulting in the bacterial cell membrane, leading to loss of intracellular contents of the cell and eventually cell death [145].

Panahandeh et al. [146] modified conventional GICs by incorporating non-spherical ZnO particles, nanoflower ZnO particles and nanorod ZnO particles. The authors evaluated flexural strength and surface hardness test using universal testing machine, while characterized the material surface analysis and crystal structure with SEM and X-radiation diffraction, respectively. The flexural strength of conventional GICs did not change after the incorporation of nanospherical and nanoflower ZnO particles, though flexural strength was slightly but not significantly increased when GIC was reinforced by nanorod ZnO particles (Table 3). A significant decrease in surface hardness was observed by nanospherical nanoflower and nanorod ZnO particles to GIC [146]. Though the mechanical properties were not enhanced by the incorporation of ZnO, NPs might be due to the fact that ZnO NPs that were incorporated are spherical in shape and therefore form agglomerates because of the Van der Waals forces. The particles stick together and cannot bond effectively with the cement matrix. These findings attributed to the initiation of cracks and resulted in weak cement [146].

In a study by Agarwala et al. [147] conventional GIC was modified by adding ZnO NPs at various concentrations and investigated the modified and unmodified GICs for shear strength, flexural strength, working time and setting time. Characterization of these cements was carried out by FTIR spectroscopy. Their study concluded that there was a marginal but statistically insignificant increase in flexural strength and shear strength. It was further demonstrated that the addition of ZnO NPs did not compromise the mechanical and handling properties of conventional GIC. It can be hypothesized that the strength was not deteriorated in the above-mentioned study due to the formation of Zn polyacrylate salt. Nevertheless, it is also noted that the strength did not get enhanced either. This might be due to the strength contributed by Zn polyacrylate being less because Zn^2+^ is a divalent ion when compared with the aluminum polyacrylate, as Al^3+^ is a trivalent ion [148].

Petromilli et al. [149] evaluated the antibacterial activity of *Streptococcus mutans* biofilm on self-cured GIC and light-cured resin-reinforced GIC after addition of ZnO NPs. This study evaluated the effect of adding low concentrations (1% and 2 wt%) of ZnO NPs on GICs. The authors demonstrated that such low concentrations did not promote the antibacterial activity against *Streptococcus mutans* [149] (Table 4). In a study conducted by Pranav P Vanajassun et al. [150] the researchers incorporated 3 wt% ZnO NPs into the conventional GIC. They found that there was no significant improvement in the compressive and shear bond strength of the experimental GIC (Table 3), whereas there was a significant increase in the antibacterial property against *Streptococcus mutans* observed after modifying the cement with ZnO NPs [150] (Table 4). It appears that there are slight differences between studies in terms of mechanical and antibacterial properties, which may be attributed to the addition of a distinct amount of ZnO NPs in GICs. Moreover, ZnO NPs are not likely to improve the mechanical characteristics of GICs, and thus may not be considered as suitable candidates for such a purpose in the real clinical environment.

### 4.2. Carbon-Based Nanostructures

Carbon-based NPs (such as graphene oxide NPs, carbon nanotubes (CNTs) and nanodiamonds) are made entirely by carbon particles [151] that exhibit a high antimicrobial activity and the efficacy of antimicrobial activity of carbon-based NPs depends on their size and surface [152]. The proposed mechanism of action involved in the antimicrobial activity of carbon-based NPs is most likely due to oxidative stress that damages the bacterial cell membrane [151].

#### 4.2.1. Graphene Oxide

Graphene is a two-dimensional (2D) material arranged in a honeycomb lattice which consists of crystalline sp2 -carbon atoms. This fundamental unit repeated itself to form a planar crystalline structure called “sheet” [153,154,155]. To enhance the properties of other materials, graphene has been studied widely due to its novel properties, such as strong mechanical properties, chemical stability, excellent biocompatibility, and antibacterial properties. Moreover, it reduces friction and wear and possesses favorable tribological properties, high conductivity, and large surface area [155,156,157,158]. Graphene can also be utilized as a substrate for the stabilization and dispersion of antibacterial NPs; for instance, Ag, Fe_3_O_4_, TiO_2_ and ZnO [159,160,161]. It can be converted into many different forms like fullerenes, a zero-dimensional (0D) nanomaterial, one-dimensional (1D) nanotube or a 3D graphite. Graphene can be found in different layers like bilayer, trilayer or multi-layers. As the number of layers increases, the properties of the material become modified. When the number of layers is greater than 10, the material shows characteristics similar to graphite [155,156,157,158].

Jingwen et al. [162] reported improved conventional GIC by adding 0–2 wt% of reduced graphene silver NPs (R-GNs/Ag). They investigated the antibacterial properties, surface microhardness, and flexural strength of conventional GICs after incorporation of R-GNs/Ag NPs. Evaluation of antibacterial properties was performed by a direct contact test (DCT) and observation through SEM, bacterial live/dead assay and XTT assay. The study results demonstrated that there was a significant decrease in the growth of *S. mutans* after the addition of R-GNs/Ag into conventional GIC. There was a slight increase in the surface microhardness and flexural strength after incorporating R-GNs/Ag at 0.1 wt%, whereas the mechanical properties were decreased by increasing the concentration of NPs. When 2 wt% concentration of RGNs/Ag were added to GIC, outstanding antibacterial properties were observed without adverse effects on their mechanical performance. Increasing the amount beyond this improved the antibacterial effect but deteriorated the surface hardness and flexure strength of the cement. Effect of adding low concentrations (0.1 wt%) of reduced graphene silver NPs R-GNs/Ag improved the microhardness and flexural strength of modified GIC, whereas these properties were adversely affected when the particles were added in higher concentrations (2 wt%) [67] (Table 3).

Recently, a new derivative of graphene family has been introduced known as fluorinated graphene (FG). This one-molecule-thick material [163] displayed many exclusive properties. Scarce literature is available on FG as it is still in its beginning era when compared to pristine graphene or graphene oxide. Nair et al. [164] stated that intrinsic strength and the Young’s modulus of graphene are higher than that of fluorinated graphene but the strength of structural steel is lower than that of fluorinated graphene. It was also found that fluorinated graphene has low friction coefficient [164]. Sun et al. [165] combined conventional GICs with fluorinated graphene at 0.5 wt%, 1 wt%, 2 wt% and 4 wt% to evaluate antibacterial properties and mechanical properties. The mechanical properties evaluated were compressive strength, flexure strength, microhardness and tribological properties. In addition, fluoride releasing properties and solubility were also investigated. It was concluded that by incorporating FG to traditional GICs boosted antibacterial, as well as mechanical and tribological properties. Moreover, it was demonstrated that when conventional GIC was combined with fluorinated graphene, color, solubility, and fluoride ion releasing properties were not compromised [166]. The findings of laboratory studies related to fluorinated graphene doped GICs indicate that such materials are likely to assist the industry in the manufacturing of strong and stable materials for the stress bearing posterior teeth.

#### 4.2.2. Carbon Nanotubes (CNTs)

Kroto et al. [167] and Ijima [168] in 1991 introduced the carbon nanotubes that are formed when graphite sheets are rolled into cylinders. There are two main types of nanotubes—single walled (SWCNTs) and multi-walled carbon nanotubes (MWCNTs). Concentric SWCNTs form the MWCNTs nanotubes. The arrangement of atoms, nanostructure morphology and the length and diameter of the tubes dictate the properties of these nanotubes [169]. CNTs have been used in many areas, especially in biomedical, dental fields and material sciences as a major reinforcing material. Carbon nanostructures have excellent biocompatibility and mechanical properties that can strengthen possibly all types of materials like polymers, ceramic, composites and metal [170]. Thus, they are a unique blend of beneficial properties like low density and ideal mechanical properties [171,172].

Many researchers have struggled with unraveling the effect of these CNTs on stem cells [173], human gingival fibroblast [174] and osteosarcoma cell lines [175]. The influence of the method of preparation of the CNTs on the level of cytotoxicity observed in both in-vitro, as well as in-vivo studies has been elaborated in various review articles [176,177,178]. Mei et al. [179] has shown that the proliferation of periodontal ligament cells was enhanced by 30% using their MWCNT incorporated GTR membrane, while gingival epithelial cells were found to exhibit less attachment. Zhang et al. [180] used surface-modified SWCNTs to reinforce a commercial resin-based dental composite and obtained encouraging results.

Goyal et al. [181] incorporated MWCNTs at two different concentrations that are 1 wt% and 2 wt% into conventional GICs. The cement was characterized by XRD, SEM, thermogravimetric analysis and differential scanning calorimetry. The mechanical properties evaluated by researchers in this study were compressive strength, wear strength and shores hardness test. Setting time, swelling property and solubility were also determined. The study found that the nanotubes had a reinforcing effect on the cement. Nonetheless, due to its black color the cement cannot be used as anterior filling material but can still be used as posterior filling cement. Mechanical properties were significantly improved in comparison with unmodified material [181]. When carbon nanotubes manufactured by electrical arc were incorporated into conventional GIC, improved compressive strength (Table 3) and resistance to erosion were observed in a study by M.R. Foroughi et al. [182]. Authors found that the composite of carbon nanotubes and bioactive glass (CNT-BG) impeded the attachment of cell line cultures. A reduced number of cells were observed on the carbon nanotubes and bioactive glass (CNT-BG) composite surface. When compared with a pure bioactive glass (BG), the absence of dense monolayer formation for glass ionomer carbon nanotubes bioactive glass (GIC-CNT-BG) composite was noticed [182]. To compare the properties of micro and NPs, Alobiedy et al. [183] incorporated 3, 5 and 7 wt% of carbon micro and NPs into the powder component of conventional GICs (Riva self-cure). They investigated biaxial flexural strength, compressive strength, Vickers microhardness and wear rate and observed relatively superior properties of GIC comprised of carbon NPs compared to the GIC containing carbon micro particles. [183].

Pani et al. [184] evaluated conventional GICs after adding combination of CNTs and silver NPs. They investigated the color stability of the GICs at time intervals of 1 h, 24 h, and 1 week. The authors displayed that the specimens fabricated without modification of NPs displayed higher color stability. However, it was concluded that the samples prepared from CNTs showed higher color stability than the samples prepared after the addition of silver NPs [184]. Undoubtedly, improved performance of CNTs modified GICs may be anticipated in the posterior teeth owing to their above-mentioned superior laboratory findings. However, their blackening effect on GICs clearly suggests that these materials cannot be used for anterior teeth restorations for the aesthetic reasons.

#### 4.2.3. Nanodiamond

Nanodiamonds (NDs) have been explored for various applications in dentistry [185]. The surface of the NDs particles is comprised of many carboxylic groups which provide a negative zeta potential to these ND particles [186]. Surface chemistry of nanodiamond NPs consists of carboxylic groups (ND-COOH), hydroxyl group (ND-OH), aldehyde (ND-CHO), carbonyl (ND-C=O), nitrogen containing groups (ND-NH_2_, ND-C·N), along with many additional groups (ND-C–F, ND-CH). Utilizing wet chemistry technique, a wide range of functional groups can be produced on ND surface. Increase in the ions release was noted when Aluminum, silicon, strontium and sodium ions present in GICs to the ND particles [187,188]. Mulder and Anderson-Small [189] modified three commercially available GICs—namely, Ketac Universal, Fuji IX and Riva Self Cure—by incorporating 5 wt% or 10 wt% ND particles in the GICs powder. The ND modified GICs exhibited a significant increase in ion release in contrast to the control GICs. However, the study’s major limitation included that the assessment of the ionic leaching from the GICs was carried only in the deionized water medium. It was proposed by the authors that further studies should be conducted about the ion movement from GICs to tooth structure. The work related to the NDs seems to be scant and at infancy stage. Further studies should be carried out so as to evaluate their effects on physico-mechanical characteristics of GICs.

### 4.3. Polymeric-Based Nanostructures

Polymers are used as NPs due to their multiplicity of compositions, structures, and properties. These polymeric NPs are incorporated into various biomaterials to fabricate composite structure suited for each specific biomedical application. They can be from natural polymers or synthetic polymers. Until now, the polymeric NPs have widely been used for drug delivery, bioimaging and biosensing application [190].

#### 4.3.1. Cellulose-Based Nanofibers and Nanocrystals

Cellulose fibers have been incorporated as a reinforcing agent into many materials, especially polymers. Investigations have reported substantial improvements in the mechanical characteristics of these materials in accordance with the amount of total fiber content [191,192]. Considering a nanotechnology approach for the development of advanced materials, nanocellulose or cellulose nanocrystals (CNC) has gained much attention recently due to its various useful features; for instance, availability in abundance, low toxic characteristics and relatively lower thermal expansion and density values [193,194]. The CNC are obtained from cellulose as rod-like nanostructures and exhibit outstanding mechanical properties. In order to achieve these structures, a controlled acid hydrolysis of naturally available cellulose is carried out, which results in the approximately 100–250 nm long nanostructures with 5–15 nm diameter. The CNC are easy to process and offer several advantages, such as their low cost, renewable nature, high mechanical properties, low density and nonabrasive nature in contrast to other commonly used inorganic fillers [194,195]. Silva et al. [196] modified the conventional GIC with CNC and the authors observed that the addition of a small amount of CNC (0.4 wt%) substantially enhanced the compressive strength, elastic modulus and diametral tensile strength up to 110%, 161%, and 53%, respectively in contrast to the control group (Table 3). The aforementioned improvements were ascribed to the uniform distribution of the CNC fibers in the cement matrix. In another study, Silva et al. [197] identified an increased fluoride release from the CNC modified GICs in addition to the improved mechanical characteristics.

Generally, watery suspension of cellulose nanofibers (CNFs) is used; therefore, performance of CNFs loaded GIC may be questioned since hydrolytic degradation of the most the dental restorative materials are widely reported in the literature [198,199,200,201]. In order to deal with such an issue, powdery CNFs have been introduced. Nishimura et al. [202] incorporated the water-free powdery CNFs into a conventional GIC and evaluated the effect of the CNFs addition on the mechanical strength and fluoride-ion release of the resultant composite. The modified GIC showed better compressive, flexural and diametral tensile strengths as compared to the unmodified GIC (Table 3). Moreover, fluoride-ion release of the modified GIC was not affected by such an addition. This class of material seems to be promising due to its positive effect on the strength and stiffness, which is suggestive of its stress bearing capability and resistance to the deformation under higher masticatory load.

#### 4.3.2. Nanochitosan

Chitosan is a natural, non-toxic, biocompatible, and biodegradable polymer obtained from crustacean shells (shrimps, crabs and lobsters). Its antimicrobial properties have been reported in the scientific literature [203]. In the pharmacology discipline, nanosized chitosan preparations have been commonly used for the delivery of different drugs [204]. Petri and coauthors evaluated the effect of chitosan on the performance of GIC [205] and observed better fluoride release and flexural strength of GIC comprising of 10 wt% chitosan. The authors attributed the aforementioned findings to the better interaction of chitosan within the GIC matrix [205]. Consequently, it is reasonable to assume that chitosan NPs may perform even better due to their relatively greater surface area and resultant enhanced interaction with the surrounding GIC matrix [206,207]. Kumar et al. [208] compared the mechanical properties and fluoride release of nanochitosan (NCH) particles modified and conventional GICs. The investigators mixed 10 wt% content of NCH with GIC and identified significantly higher compressive and flexural strength values compared to the control group (Table 3). Moreover, wear resistance of experimental GIC was enhanced possibly due to better bonding of NCH with the GIC matrix. In addition, the experimental GIC showed significantly higher fluoride release compared to the control GIC up to 1 week. It appears that NCH addition to GIC is likely to enhance the anti-cariogenic potential, as well as mechanical characteristics; hence, a long survival rate of GIC restorations in the load bearing posterior dentition may be expected.

### 4.4. Inorganic-Based Nanostrcutures

Inorganic NPs are particles that do not contain carbon atoms in them. They include zirconia, silica, hydroxyapatite, barium sulfate, tertium fluoride and silica clay. In recent years, inorganic NPs have engrossed researchers’ attention due to their physical properties; for instance, optical, magnetic and chemical properties such as inertness, stability, and ease of functionalization [209].

#### 4.4.1. Hydroxyapatite (HA)

HA is a natural mineral component of tooth enamel that grows in hexagonal crystals. It is a white, naturally occurring form of the mineral calcium apatite, i.e., calcium, phosphorous, and oxygen. A major part of the bone, tooth enamel, and dentine structure in humans is composed of hydroxyapatite comprising phosphate and hydroxyl ions, which can form a bond with bone structure and implants; hence, it can allow osseointegration [210,211]. The most striking feature of the material is its biocompatibility, as it is a natural constituent of human dental and skeletal structures. Therefore, several studies are conducted in which HA has been incorporated in dental restorative materials like GICs [74,212,213,214,215]. Results from the studies have shown better biocompatibility of the GIC incorporated with HA and improved mechanical properties [216]. In restorative dentistry, HA has shown encouraging benefits in intrinsic radiopacity and hardness similar to that of natural tooth and biocompatibility [63,217]. This naturally occurring mineral can be converted to NPs of HA by many different methodologies—for example, co-precipitation, sol-gel synthesis and wet chemical preparation [218]. After the incorporation of HA in GICs, researchers evaluated the compressive and diametral tensile strength, [159] flexural strength, [157] toughness, bonding and fluoride-release properties [219]. Arita et al. [220] evaluated the effects of the incorporation of HA whiskers on the flexural strength and microstructure of conventional GIC at 10–28 wt%. These properties were evaluated at wet and dry conditions. It was concluded that either in whiskers or granules forms, the addition of HA improved the flexural strength and microstructural properties of GIC [219] (Table 3).

Noorani et al. [220] performed a study in which nano-hydroxyapatite-silica was incorporated in conventional GIC (HA-SiO_2_-GIC), and cytotoxic effects were then evaluated. They investigated these effects on human Dental Pulp Stem Cells (DPSC). The authors used conventional GICs—namely, Fuji IX and Fuji II LC against nano-hydroxyapatite-silica incorporated GIC (HA-SiO_2_-GIC) and prepared seven serial concentrations. An inverted phase-contrast microscope was used to observe the morphology of dental pulp stem cells, and MTT assay was utilized to determine the cell viability at 24 h and 72 h. The authors concluded that the most biocompatible material in terms of its cytotoxicity was Fuji IX tailed by HA-SiO_2_-GIC, whereas Fuji II LC demonstrated the least biocompatibility. A favorable biological response was confirmed by HA-SiO_2_-GIC, which is comparable to that of conventional GIC. Nano-hydroxyapatite-silica included GIC HA-SiO_2_-GIC and may be reflected as favorable potential restorative material, though its possible use needs to be authenticated by further in vitro and in vivo investigations [220]. To overcome the disadvantages of GIC without losing their favorable clinical advantages, Moshaverinia et al. [221] conducted a study in which they added nano-hydroxyapatite and fluorapatite. The authors utilized a ratio of 20:1 *w/w* for the glass powder having HA and glass powder having FA. They evaluated compressive strength, diametral tensile strength, and flexure strength. In conclusion, the addition of nano-HA and FA into GICs enhanced the mechanical strength compared to traditional GICs [222]. The benefits of HA nanostructures alone in terms of mechanical properties are well evident. However, their application, along with silica, causes a great concern as their combination compromised the biocompatibility of some GICs. It is proposed that further studies in this area should be designed to explore this tooth matching material for future clinical applications.

#### 4.4.2. Zirconia

ZrO_2_ is a biomaterial having natural white color, excellent biocompatibility, corrosion resistance, high strength, chemically stability, excellent fracture toughness, resistance to cracking, low thermal and high ionic conductivity [223]. Due to its high strength, the processing of zirconium particles is complicated. NPs of zirconium can be used as nanopowders filling [224], nano-coating [225] and sintering raw materials [226]. The nanopowder of the ZrO_2_ can be incorporated into various dental materials and can be used as a scaffold in tissue engineering to enhance their mechanical strength. The addition of ZrO_2_ nanopowders into dental materials will significantly increase the flexural strength, fracture toughness and shear bond strength of these materials [227]. In dentistry, over the last many years, zirconia has also been used as dental abutments, implants, crowns and bridges in posterior restorations [228,229]. Recently, to closely match colors of human teeth, zirconia has been established with enhanced translucency that has high flexural strength (900–1400 MPa) and fracture toughness (6 MPa) [230].

GIC was modified with HA-zirconium HA/ZrO_2_ NPs by Gu et al. [231]. Variable volume percentages (4, 12, 28 and 40 vol%) were incorporated in a conventional GIC. HA/ZrO_2_-GIC composite was immersed in distilled water for time periods of 1 day and 1 week and then tested for their mechanical strength. They found that within the GIC matrix, the HA/ZrO_2_ and glass particles were uniformly dispersed on SEM examination. Authors found that diametral tensile strength, compressive strength and surface microhardness of HA/ZrO_2_-GIC were improved when compared with the conventional GIC (Table 3). They demonstrated that mechanical properties also increase as the soaking time increases. This improved final strength of the cements might be because of the continuous formation of aluminum salt bridges. Moreover, by increasing the soaking time, ZrO_2_ did not dissolve. To evaluate the genotoxic effect of zirconium particles on human gingival fibroblasts (HGFs), Laiteerapong et al. [232] formulated a novel GIC containing zirconia macroparticles (MPs) and NPs. The authors used H2AX fluorescent assay to evaluate genotoxic effect of zirconium particles on HGFs. Both conventional and modified GIC had no genotoxic effect on HGFs [232].

A study was conducted by Gjorgievska et al. [139] with the aim of improving mechanical properties of conventional GICs and evaluated the impact of addition of Al_2_O_3_, ZrO_2_ and TiO_2_ NPs at 2, 5 or 10 wt%, respectively. The researchers found that ZrO_2_ and TiO_2_ NPs increased the compressive strength, whereas they demonstrated that the cement became weak by the addition of Al_2_O_3_. Spectrometric analysis displayed that NPs did not release a noticeable number of ions (Al, Zr or Ti), which makes them appropriate for clinical use. Sajjad et al. [233] utilized one-pot technique for the synthesis of nano zirconia–silica–hydroxyapatite (nano ZrO_2_-SiO_2_-HA). They characterized the materials using energy dispersive X-ray (EDX), dot mapping, FTIR, SEM and transmission electron microscopy (TEM). Compressive strength, flexural strength and surface roughness were evaluated. Significant increase in the flexural and compressive strengths was observed after addition of 5 wt% nano ZrO_2_-SiO_2_-HA in conventional GICs (Table 3). Moreover, surface roughness profile of modified GIC was similar to conventional GICs. Hence, the GICs modified with nanoZrO_2_-SiO_2_-HA has favorable properties, and these materials can be used for permanent restorations in the high stress bearing areas [233].

It appears that the effect of ZrO_2_ on the performance of GICs has been investigated in combination with other ingredients—namely, Al2O_3_, TiO_2,_ SiO_2_ and HA. Overall, the combination seems to be effective with mechanical tastings, except with Al_2_O_3,_ which led to weak cement. Therefore, researchers must consider this issue in future studies.

#### 4.4.3. Halloysite Nanotubes

Naturally occurring aluminosilicate mineral materials are basically the Halloysite nanotubes (HNTs) and are obtained from clays. Due to their high aspect ratio and hollow core structure, these materials are considered for the filling of drugs within the inner lumen, as well as for the material reinforcement [232,233,234,235,236]. Moreover, they are relatively economical as compared to the carbon nanotubes [237]. This class of material has been widely investigated in the dental materials. A research group incorporated 8 wt% HNTs into resin-based dental composites and observed either inferior mechanical properties or no significant effect as compared to the control group [238,239]. However, lower concentrations (<5 wt%) of HNTs significantly improved the mechanical properties [240]. In adhesive resins and denture base resins, moderate concentrations of HNTs increased their hardness value [241,242]. In a study by Holder et al. [243], 5 wt% HNT was incorporated into GIC and physico-mechanical and fluoride release characteristics of both the conventional and modified GICs were evaluated. The addition of 5 wt% HNTs showed greater compressive strength (187 MPa) as compared to the unmodified GIC (140 MPa) (Table 3). Moreover, the hardness and wear resistance of the modified GIC was also greater than the unmodified GIC by 11% and 22%, respectively. However, HNT-GICs did not show any significant change in the diametral tensile strength, and their fluoride release activity was decreased by 14% over 4 weeks’ time. The authors suggested that reinforcing the existing GICs with HNT may provide suitable restorative materials for the load bearing situations. The findings of HNTs modified GICs are promising in terms of physico-mechanical characteristics; hence, a better clinical performance may be expected as compared to the traditional GICs. However, data are inadequate and warrant further investigation of this material.

#### 4.4.4. Montmorillonite Nanoclays

Polymer-based materials are being frequently reinforced with the low quantities of montmorillonite (MMT) nanoclay (1–5%). The most common polymers which have been reinforced with the nanoclay dispersions include nylon [244], epoxy resins [245], polycaprolactone [246], polystyrene [247], polyethylene [248] (polymethyl methacrylate [249], chitosan [250], polyamide [251], PAA based composites [252] and polyurethane [253]. Polymer grade (PG) montmorillonites (PGV and PGN) are the high purity minerals which are mainly based on alumina–silicate and are commonly incorporated in the hydrophilic polymers namely polysaccharides and such as polyvinyl-alcohols. The composition of glass powder and polyacid mainly affect the mechanical properties of GICs [254]. Uniform dispersion of nanoclay particles in the polymeric matrix of GIC can improve its strength, and the selection of nanoclay for PAA plays a vital role [255]. Fareed and Stamboulis [256] confirmed the dispersion of montmorillonite nanoclays (PGV and PGN) in PAA in GICs and same authors, in other studies [257,258] evaluated the effect of 2 wt% polymer-grade montmorillonite (PGV and PGN nanoclay) on a commercial GIC (Fuji-IX) glass. The experimental GICs exhibited higher mechanical characteristics than Fuji-IX cement and met the ISO standard requirement. Moreover, exfoliation and dispersion of nanoclay in the GIC matrix’ was observed under TEM examination. The authors of above studies suggested that the addition of nanoclays in GICs may possibly aid the development of mechanically better dental cements for the restoration of posterior teeth. The effect of Montmorillonite nanoclays on the GICs seems to be positive, as reported by a group of authors described above. Hence, their superior clinical performance is expected.

#### 4.4.5. Chlorhexidine Hexametaphosphate

The efficacy of chlorhexidine (CHX) antimicrobial agent against both Gram-negative and Gram-positive bacteria is well known. It acts by disrupting the cell membrane, and it does not allow the resistance of bacteria, which is common with the extensive use of antibiotics [259]. CHX digluconate is readily available and is one of the frequently used soluble salts. In biomaterials science, some materials are soaked in CHX digluconate solutions in order to achieve their antimicrobial properties [260,261]. Sometimes, CHX diacetate in a dry crystalline powder form is added to the materials to enhance their antibacterial performance [262,263,264].

Barbour et al. [265] synthesized novel antimicrobial NPs by combining sodium hexametaphosphate and solution of CHX digluconate. An instant reaction led to a stable colloid comprised of NPs of size 20−160 nm with a highly negative charge NPs (−50 mV). The authors reported that NPs are capable of bonding with glass, titanium, and an elastomeric wound dressing specimen and allow gradual release of soluble CHX up to 50 days. Moreover, these NPs exhibit effectiveness against *Staphylococcus aureus* and *Pseudomonas aeruginosa* under planktonic and biofilm conditions. Hook et al. [266] formulated experimental GICs by replacing their powder content with novel NPs of CHX hexametaphosphate. The findings of their study indicate that the CHX was released from the GICs throughout the study period. The addition of up to 10% NPs did not affect the fluoride release; however, the replacement of GIC powder with 10 and 20% NPs caused a reduction in the diametral tensile strength values (Table 3). The authors suggested that these novel NPs may aid the development of new antimicrobial dental restorative materials. Likewise, Hosida et al. [125] evaluated the influence of hexametaphosphate NPs addition on the antimicrobial properties, fluoride (F) release, enamel demineralization and physico-mechanical behavior of GICs. The GICs containing 9 and 12% hexametaphosphate NPs showed the highest levels of fluoride release, improved antibacterial properties (Table 4) and reduced loss of mineral. However, the physico-mechanical properties were compromised with the addition of hexametaphosphate NPs in contrast to the control group. The new class of materials can be employed for the patients with high caries risk and low stress-bearing regions of the oral cavity owing to their superior antimicrobial properties and inferior mechanical properties.

#### 4.4.6. Ytterbium Fluoride and Barium Sulfate

Ytterbium belongs to the lanthanide series in Periodic Table. The atomic number of ytterbium is (z = 71) and when forming a fluoride glass ytterbium, refractive index is approximately 1.5. It is a very biocompatible, ductile, and heavy metal that slowly reacts with water and is used in imaging examination. To impart radiopacity, ytterbium trifluoride (YbF_3_), barium sulphate and bismuth oxide have been incorporated in GIC without significantly compromising the properties of the material [267,268]. When barium hydroxide reacts with sulfuric acid, then barium sulphate (BaSO_4_) is formed. Depending on formulation and condition, the barium sulphate can be moulded in several structures like planar, starred, or spherical structures. Planar barium sulphate has a high light-scattering property and demonstrates high lubricity [269]. In restorative materials, radiopacity is an important property. It is stated that radiopaque GICs are often less translucent than non-radiopaque GICs. More aesthetic GICs are likely to develop after incorporation of radiopaque NPs into these cements. In therapeutic biomedical applications, barium sulfate particles have formerly been inspected as radiopacifiers [270].

To improve radiopacity of GICs, Prentice et al. [271] studied conventional GICs by incorporating BaSO_4_ 1–25% (m/m) having less than 10 nm size and YbF_3_ NPs having a size of 25 nm. The addition of BaSO_4_ (2 m%) and YbF_3_ (1 m%) NPs decreases the initial setting and working time of the GIC. At higher concentrations, however, the effect was upturned. When 2–5% (m/m) of YbF_3_ or BaSO_4_ was added to conventional GICs, working time was found to be shortest, whereas at 10% (m/m) addition of BaSO_4_ initial setting time was found to be shortest [268]. Furthermore, decreased compressive strength was observed after the addition of BaSO_4_ and YbF_3_ to the cement (1% of powder) (Table 3). The decrease in the working and setting time of these GIC is due to the interaction of these NPs to the initial gelation reaction of GIC matrix. There was a marked interference between the normal GIC setting reaction and BaSO_4_ which led to a reduction in strength that was significant even at 1% addition of BaSO_4_ to the powder. This study pointed out that the incorporation of YbF_3_ and BaSO_4_ NPs into GIC significantly reduced its surface hardness and 24 h compressive strength (Table 3). Lastly, it was concluded that YbF_3_ and BaSO_4_ (low concentrations) accelerates curing reaction of GICs, but at higher concentrations of BaSO_4_ had reverse effects [271]. This class of materials seems to be poor as their addition into the GICs led to a significant reduction in the surface hardness and strength. Hence, this addition may not provide suitable materials for the posterior restorations.

**Table 2:** Advantages, limitations and clinical significance of various nanostructures in GICs.

Nanostructure Type	Advantages	Limitations	Significance	References
Zinc Oxide NPs	Strength either did not change or marginally increased 3 wt% of ZnO NPs promoted the antibacterial activity	Surface hardness significantly decreased 2 wt% of ZnO NPs did not promote the antibacterial activity	ZnO NPs are not likely to improve the mechanical characteristics of GICs, hence they may not be considered as suitable candidates for posterior restorations	[148,149,150]
Reduced graphene silver NPs	Antibacterial activity was increased with 2 wt% of Reduced graphene silver NPs	Surface hardness and mechanical properties were deteriorated significantly with 2 wt% of reduced graphene silver NPs	Reduced graphene silver NPs are likely to prevent the secondary caries after their incorporation in GICs, however, they may compromise the performance of GICs in permanent posterior teeth	[162]
Fluorinated graphene NPs	Boost antibacterial, as well as mechanical and tribological properties of GICs. Do not compromise the color, solubility and fluoride ion releasing properties of GICs		Fluorinated grapheme NPs could be suitable candidates for the reinforcement of GICs for posterior restorations	[165,166]
Multi-walled carbon nanotubes (MWCNTs)	MWCNTs reinforced the cement	MWCNTs caused blackening of cement	Cannot be used in anterior teeth due to the blackening of cement	[181]
Nanodiamond (ND)	The ND modified GICs exhibited a significant increase in ion release	The major limitation included that the assessment of the ionic leaching from the GICs was carried only in the deionized water medium	This nanostructure may aid the remineralization of tooth structure	[189]
Titanium NPs	An improved fracture toughness, flexural strength and compressive strength with addition of 3 and 5 wt% TiO_2_ NPs were observed. Surface coating of TiO_2_ NPs on orthodontic wires and brackets showed antibacterial activity	Reduction in fracture toughness, flexural strength and compressive strength was observed with addition of 7 wt% TiO_2_ NPs Decreased Surface microhardness with 5 and 7 wt% TiO_2_ NPs was identified	Up to 3 wt% addition could make the GICs suitable for permanent posterior fillings Accumulation of bacteria may be avoided on the orthodontic wires and brackets which in turn prevent the infection and secondary caries	[138,140,141]
Silver NPs	No cytotoxic affect Higher compressive strength Higher surface microhardness Higher dentin micro shear bond strength Significant antibiofilm activity		Could be suitable candidate for load bearing posterior dentition and reduce the likelihood of secondary caries	[115,116,119]
Copper NPs	Positive antibacterial activity		Reduce the likelihood of secondary caries	[127]
Magnesium oxide NPs	Positive antibacterial effect Increased compressive, diametral tensile and shear bond strength Cost-effective	Increased setting time	Could be suitable candidate for load bearing posterior dentition and reduce the likelihood of secondary caries However, moist environment may compromise the survival rate due to increased setting time	[133,134]
Cellulose-Based Nanofibers and Nanocrystals	A small amount of CNC (0.4 wt%) substantially enhanced the compressive strength, elastic modulus and diametral tensile strength of GIC Increased fluoride release from GICs	Watery suspension of cellulose nanofibers (CNFs) may lead to the degradation of GICs	Its positive effect on the strength and stiffness may provide stress bearing capability to GIC restorations	[197,198]
Nanochitosan (NCH)	NCH significantly increased the compressive and flexural strength values of GICs NCH enhanced the fluoride release and wear resistance of GICs		NCH addition to GIC is likely to enhance the anti-cariogenic potential as well as mechanical characteristics. Long survival rate of GIC restorations in the load bearing posterior dentition may be expected	[205,208]
Hydroxyapatite (HA)	The addition of HA improves the flexural strength and microstructural properties	Their application along with silica causes a great concern as their combination compromised the biocompatibility of some GICs	The benefits of HA nanostructures alone in terms of mechanical properties are well evident, hence they could be considered as suitable materials for the restoration of posterior teeth	[220,221,222]
Halloysite Nanotubes (HNTs)	The addition of 5% HNTs showed greater compressive strength The hardness and wear resistance were also increased	Fluoride release was decreased	The findings of HNTs modified GICs are promising in terms of physico-mechanical; hence a better clinical performance may be expected as compared to the traditional GICs	[242]
Montmorillonite (MMT) nanoclay	Uniform dispersion of nanoclay particles in the polymeric matrix of GIC was evident Montmorillonite (MMT) nanoclay improved the mechanical properties of GICs		Superior clinical performance is expected due to their enhanced mechanical properties.	[158,159,160,161,162,163,164,165,166,167,168,169,170,171,172,173,174,175,176,177,178,179,180,181,182,183,184,185,186,187,188,189,190,191,192,193,194,195,196,197,198,199,200,201,202,203,204,205,206,207,208,209,210,211,212,213,214,215,216,217,218,219,220,221,222,223,224,225,226,227,228,229,230,231,232,233,234,235,236,237,238,239,240,241,242,243,244,245,246,247,248,249,250,251,252,253,254]
Chlorhexidine Hexametaphosphate NPs	CHX was released from the GICs The addition of up to 10% NPs did not affect the fluoride release 9 and 12% hexametaphosphate NPs showed the highest levels of fluoride release and improved antibacterial properties.	The replacement of GIC powder with 10 and 20 wt% NPs caused a reduction in the diametral tensile strength values The physico-mechanical properties were compromised with the addition of hexametaphosphate NPs	This new class of materials could be employed for the patients with high caries risk and low stress-bearing regions	[265,266]

**Table 3:** Effect of the incorporation of various nanostructures on the mechanical strength of GICs.

Nanostructure Type	Strength Type	Effect	Clinical Relevance	References
Zinc Oxide NPs	Compressive strength andShear Strength	No effect on compressive and Shear Strength with 3 wt%	May not perform successfully in the load bearing posterior dentition	[150]
Zinc Oxide NPs	Flexural strength	No effect on flexural strength with 5 wt%	May not perform successfully in the laod bearing posterior dentition	[146]
Reduced Graphene Silver NPs	Flexural strength	Increased with 0.1 wt% Decreased with 2 wt%	May perform successfully in the load bearing posterior dentition	[162]
Carbon Nanotubes	Compressive strength	Increased with 1–2 wt%	May perform successfully in the laod bearing posterior dentition	[181]
Hydroxyapatite Whiskers/Granules	Flexural strength	Increased with 10–28 wt%	May perform successfully in the laod bearing posterior dentition	[219]
Ytterbium Flouride and Barium Suphate	Compressive strength	Decreased with 1 wt%	May not perform successfully in the laod bearing posterior dentition	[270]
Titanium NPs	Flexural strength and Compressive strength	Increased with 3–5 wt% Decreased with 7 wt%	May perform successfully in the laod bearing posterior dentition	[131]
Hydroxyapatite-zirconium NPs	Diametral tensile strength and Compressive strength	Increased with 4, 12, 28 and 40 vol%	May perform successfully in the laod bearing posterior dentition	[230]
Nano Zirconia-Silica Hydroxyapatite	Flexural strength and Compressive strength	Increased with 5 wt%		[233]
Silver NPs	Compressive strength, Micro shear bond strength and Flexural strength	Compressive strength Increased with 0.1 and 0.2 wt% Flexural strength increased with 0.2 wt%	May perform successfully in the laod bearing posterior dentition	[118,119]
Cellulose Nanocrystals	Diametral tensile strength and Compressive strength	Increased with 0.4 wt%	May perform successfully in the laod bearing posterior dentition	[198]
Cellulose Nanofibers	Flexural strength, Diametral tensile strength and Compressive strength	Increased with 2–8 wt%	May perform successfully in the laod bearing posterior dentition	[201]
Nanochitosan	Flexural strength and Compressive strength	Increased with 10 wt%	May perform successfully in the laod bearing posterior dentition	[[208]
Magnesium oxide NPs	Diametral tensile strength, Compressive strength and Shear bond strength	Increased with 1 wt%	May perform successfully in the laod bearing posterior dentition	[134]
Halloysite Nanotubes	Compressive strength	Increased with 5 wt%		[243]
Chlorhexidine Hexametaphosphate NPs	Diametral tensile strength	Decreased with 10–20 wt%	May not perform successfully in the laod bearing posterior dentition	[266]

**Table 4:** Effect of the incorporation of various nanostructures on the antibacterial activity of GICs.

Nanostructure Type	Bacteria Type	Effect	Clinical Relevance	References
Zinc Oxide NPs	*Streptococcus mutans*	No effect was observed with 1–2 wt%	May not prevent secondary caries	[147]
Zinc Oxide NPs	*Streptococcus mutans*	Increased antibacterial activity with 3 wt%	Likely to prevent the secondary caries	[150]
Reduced graphene silver NPs	*Streptococcus mutans*	Significant decrease in growth of bacteria was observed with 2 wt%	Likely to prevent the secondary caries	[162]
Titanium NPs	*Streptococcus mutans*	Strong antibacterial effect was observed with 3 wt%	Likely to prevent the secondary caries	[124]
Silver NPs	*Streptococcus mutans*	Significant antibiofilm activity was observed with 6–24 μg Ag/capsule	Likely to prevent the secondary caries	[119]
Copper NPs	*Streptococcu mutans* and *Streptococcus sanguinis*	Antibacterial activity was observed with 2–4 wt%	Likely to prevent the secondary caries	[127]
Magnesium oxide NPs	*Streptococcus mutans* and *Streptococcus sobrinus*	Effective antibacterial activity was observed with 1 wt%.	Likely to prevent the secondary caries	[133]
Chlorhexidine Hexametaphosphate NPs	*Streptococcus mutans, Lactobacillus acidophilus* and *Actinomyces israelii*	Antibacterial activity was observed with 9–12 wt%	Likely to prevent the secondary caries	[125]

## 5. Challenges and Limitations

In general, most of the attempts by the researchers for the reinforcement of GICs with nanostructures seem to be at the experimental stage, and long-term clinical trials are not frequently conducted. The possible reasons include lack of funds for the clinical research, or a lack of interest among clinicians, as alternative tooth-colored successful restorative materials are also available. However, some valuable features of GICs—for instance, anticariogenic properties and tooth bonding potential—are far better than the resin composite restoratives. Considering these advantages, clinical scientists should be provided with research grants to explore the performance of nanostructures modified GICs in a clinical environment that may ultimately aid the development of better materials. Despite the favorable effects of nanotechnology, the applications of dental nanomaterials have also caused serious concerns among researchers about their potential toxicity to the human body. Although humans are exposed to various nanoscale materials during routine life, the new emerging field of nanotechnology has some unwanted effects^.^ [272]. Because of their small size, NPs find their way easily into the human body and cross the various biological barriers, reaching the most sensitive tissues [273]. Scientists have proposed that NPs of sizes less than 10 nm act similar to a gas and can enter human tissues easily and may disrupt the cell’s normal biochemical environment [274]. Animals and human studies have shown that after inhalation and through oral exposure, NPs are distributed to the liver, heart, spleen, and brain in addition to lungs and gastrointestinal tract [275,276,277,278]. Due to high surface area and surface energy, NPs are more toxic to human health in comparison to large-sized particles of the same chemical substance. It is usually suggested that toxicities are inversely proportional to the size of the NPs [278]. For instance, several toxic effects of TiO_2_ NPs on the human body are well evident in the scientific literature [279,280]. In addition, the cellular toxicity of ZnO nanostructures has also been reported in in-vitro studies [281]. In a study conducted by Sharma et al. [282] DNA damaging potential of ZnO nanostructures was demonstrated in human epidermal cells. Even at low concentration, ZnO NPs demonstrated genotoxic in human epidermal cells. It was postulated that this might be due to lipid peroxidation and oxidative stress. Recently, Song et al. [283] evaluated the in vitro cytotoxicity of ZnO NPs on the human epithelial colorectal adenocarcinoma (Caco-2). They utilized Reactive oxygen species (ROS), superoxide dismutase (SOD) and glutathione (GSH) assay to explore the oxidative damage of Caco-2 cells and reported that ZnO NPs are highly cytotoxic for the human cells. Even though these NPs can easily cross physiological barriers and cause an adverse effect, Semyari et al. [284] proved the improved biocompatibility of cements containing zirconia (ZrO_2_) NPs [284]. Moreover, in the same study it was also found that their free radical concentration was increased, resulting in damage to the cells when ZrO_2_ was incorporated into the cement.

Table 5 summarizes the toxic effect of various nanostructures used in dentistry. The current literature indicates that the nanostructures can deposit in various organs including liver, lungs, kidneys, and hearts of animals [285,286]. It is certainly understandable that the blood–brain barrier can prevent the entry of most of the substances in the brain. On the contrary, various nanostructures have easily passed through the blood-brain barrier into the brain, owing to their small sizes and greater surface activities [287,288]. These findings highlight that the central nervous system could be easily targeted by the nanostructures and as a result a series of pathogenic events may occur. Therefore, potential risks to the human body related with nanomaterials should always be considered. However, currently, the standards for the monitoring and governing of nanomaterials for dental applications are lacking. Moreover, no effective strategy for the assessment of long-term exposure risks to patients is documented. This warrants the establishment of the standards for the development of safe materials in terms of handling and in situ.

## 6. Critical Appraisal and Future Trends

The effects of various nanostructures on the laboratory-based performance of GICs seems to be widely researched by investigators; however, such attempts lack appropriate approaches. For instance, some standards already exist as guidelines for the in-vitro testing of GICs; however, a majority of researchers are not following such well-established protocols. Consequently, the comparison of results may not be made among researchers and thus render the data less meaningful. In addition, none of the research group has considered the long-term storage of nanostructured modified GIC specimens in clinically simulated conditions; consequently, it is difficult to predict the survival rate of GIC restorations during the actual clinical performance.

Although several studies report either enhanced or declined physical, mechanical and antibacterial properties of nanostructured modified GICs, the factors which are responsible for such changes in the properties are not fully investigated. Therefore, we propose that further structural and chemical analyses of modified GICs should be conducted. For example, dispersion of nanostructures in the GIC matrix should be evaluated using SEM or TEM which may highlight whether the nanostructures are uniformly distributed or have made random agglomerates across the matrix. Moreover, researchers should evaluate the chemical interaction of nanostructures with GIC matrix by the FTIR in order to correlate the physico-mechanical properties. Furthermore, lack of consensus regarding antibacterial testing methods is well evident among researchers, and hence it is proposed that guidelines for this important aspect should be reviewed and standardized by the academy of dental materials or other relevant authorities.

To date, a variety of nanostructures have been synthesized using top-down and bottom-up approaches. Such nanostructures have been widely incorporated in the existing dental biomaterials to improve their physical, mechanical, antimicrobial and handling characteristics, and, eventually, the clinical performance. It is certainly understandable that such attempts are promising as far as laboratory-based studies are concerned. However, there is a lack of clinical studies, and thus, more clinical data are warranted in order to develop the improved materials. While reviewing the literature about the GICs in particular, it is well evident that incorporating most of the nanostructures enhances their mechanical strength and antibacterial activity against dental caries causing bacteria when added up to a certain percentage. Among these nanostructures, some of them lead to inferior properties of GICs when added in excess.

Nevertheless, the findings are encouraging, but most of the GIC related data are based on experimental research. It is difficult to predict their clinical performance, as inconsistency is common among investigators in terms of testing methodologies. Moreover, clinical studies are being rarely considered by clinical scientists. Therefore, keeping the above facts in view, standardization of testing methods should be carried out and must be followed across the research community. Furthermore, clinical scientists should consider the long-term prospective studies on patients to attain durable restorative material with improved aesthetic and antibacterial properties.

## Figures and Tables

**Table 1:** Brief Overview of Nanostructures and their Dental Applications.

Nanostructures	Characteristics and Applications	References
Nanorods	HA nanorods have exhibited self-assembly properties HA nanorods addition into the dental adhesives significantly increase their bulk mechanical properties and micro-shear strength	[21,22]
Nanospheres	Mimic nanoscale processes inherent in natural tooth development Provide scaffolding for the initial enamel apatite crystals nucleation and growth Provide therapeutic effect by releasing growth factors over a prolonged period	[21,22,23,24]
Nanotubes	Titanium oxide nanotubes accelerate the kinetics of HA formation, which employed mainly in bone-growth applications for dental implant coatings Modified single-walled carbon nanotubes (SWCNTs) improve the flexural strength of resin-based composites Carbon nanotube (CNT) coating on titanium plates increases alkaline phosphatase (ALP) activity and osteoblastic proliferation	[25,26,27]
Nanofibers	Nanofibrillar silicate crystals reinforce dental composites Improve the physical properties of composites by hindering the crack propagation	[28,29,30,31]
Dendrimers or dendritic copolymers	Increase the degree of bond conversion thus, improving the strength of the resultant structure	[32,33,34]
Nanopores	A nanopore range up to 100 nm in posterior composite improves its wear resistance Alumina particles as nanopore increase the mechanical interlocking between fillers and matrix of composite restoratives without the need for chemical bond	[35,36]
Quantum dots (QD)	Possess distinctive optical properties and fluorescence potential Provide excellent aesthetics of the composite restorative material	[37]
Nanoshells	Repair periodontal defects as they help in controlled site-specific drug delivery into the periodontal tissue Fatal for tumour cells in oral cancer patients while normal cells remain unaffected	[38]
Liposomes	Exhibit antimicrobial activity and prevent dental caries	[39,40]
Fullerenes	Enhance photocatalytic efficiency when added in combination with zinc oxide NPs	[41,42]
Nanowires	Used as a scaffold in tissue engineering for the treatment of carious lesions and enamel repair HA nanowires improve mechanical properties of dental resin-based composites	[23,43]
Nanobelts	Used in biomimetic dentistry	[44,45]
Nanocapsules	Polyurethane nanocapsules improve bond durability and strength of dental adhesives Help during acid etching technique	[46,47,48]

**Table 5:** Toxic effects of various nanostructures used in dentistry.

Nanostructures	Toxic Effect	References
Aluminum oxide	Disturbed the cell viability Altered mitochondrial function Increased oxidative stress Altered expression of the blood brain barrier (BBB)	[289]
Gold	Relatively safe Toxicity is dependent on the type of toxicity assay, cell line, and physical/chemical properties, dose, side chain (cationic) and the stabilizer	[290,291,292,293]
Copper oxide	Toxic effects on the liver, kidney and spleen in experimental animals Genotoxic and cytotoxic Cell membrane integrity disturbed Prompting oxidative stress	[294,295,296]
Silver	After exposing the rats to these NPs, detected in various organs, including lungs, spleen, kidney, liver, and brain. Cell viability disturbed Reactive oxygen species (ROS) generated Leakage of lactate dehydrogenase (LDH) Dose-dependent cytotoxicity	[297,298,299]
Zinc oxide	Oxidative stress increased Damaged cell membrane Complete cell death Cell morphology changed Damaged DNA In human hepatocytes, and embryonic kidney cells alteration in mitochondrial activity Genotoxicity	[300,301,302,303]
Iron oxide	Store in the liver, spleen, lungs, and brain Ability to cross BBB Cell Inflammation, lysis and reduced cell viability reduced Blood coagulation system impaired	[304,305]
Titanium oxide	Genotoxicity Damaged DNA Inflammation of lungs Increased oxidative stress Increased toxic effects on immune function, liver, kidney, spleen, myocardium, glucose, and lipids homeostasis in experimental animal	[306,307,308]
Carbon-based nanomaterials	Size-dependent cytotoxicity Genotoxicity in the form of micronucleus formation, chromosomal damage, and DNA strand breakage.	[309,310,311]
Silica	Increased ROS Increased oxidative stress Increase LDH Increased malondialdehyde after treating human bronchoalveolar carcinoma cells at a dosage range of 10–100 µg/mL	[312,313,314]
NPs of polymeric materials	Non-toxic, non-immunologic and non-inflammatory Did not activate neutrophils However, toxicity towards human-like macrophages after surface coating was observed	[315]

## Data Availability

Not applicable.

## Abbreviations

glass ionomer cements	GICs
polyacrylic acid	PAA
Hydroxyapatite	HA
single-walled carbon nanotubes	SWCNTs
carbon nanotube	CNT
alkaline phosphatase	ALP
quantum dots	QD
atraumatic Restorative Treatment	ART
nanoparticles	NPs
reactive oxygen species	ROS
resin-modified glass ionomer	RMGIs
nano-hydroxyapatite	Nano-HA
nanoflouroapatite	Nano-FA
scan electronic microscope	SEM
weight%	Wt%
copper nanoparticles	CNu
Transmission electron microscopy	TEM
fourier transform infrared	FTIR
zero-dimensional	0D
one-dimensional	1D
nano-hydroxyapatite-silica incorporated GIC	HA-SiO_2_-GIC
inductively coupled plasma-optical emission spectrometry	ICP-OES
reduced graphene silver	R-GNs/Ag
direct contact test	DCT
fluorinated graphene	FG
multi-walled carbon nanotubes	MWCNTs
guided tissue regeneration	GTR
bioactive glass	BG
nanodiamonds	NDs
cellulose nanocrystals	CNC
cellulose nanofibers	CNFs
nanochitosan	NCH
dental Pulp Stem Cells	DPSC
halloysite nanotubes	HNTs
montmorillonite	MMT
polymer grade	PG
chlorhexidine	CHX
lactate dehydrogenase	LDH
blood brain barrier	BBB
hydroxyapatite zirconium	HA/ZrO_2_
volume%	Vol%
human gingival fibroblasts	HGFs
energy dispersive X-ray	EDX
nano zirconia–silica–hydroxyapatite	nano ZrO_2_–SiO_2_–HA
nanoionomeric hybrid resin-modified glass ionomer	NHRMGI
